# Supplementary material for: Dimethyl Sulfoxide as a Biocompatible Extractant for Enzymatic Bioluminescent Toxicity Assays: Experimental Validation and Molecular Dynamics Insights
Source: Toxics. 2025 Nov 30;13(12):1038. doi: 10.3390/toxics13121038 (PMC12736966; doi:10.3390/toxics13121038)
Supplement: Supplementary file 1 [file toxics-13-01038-s001.zip › toxics-4000839-supplementary.pdf]

Supplementary Material

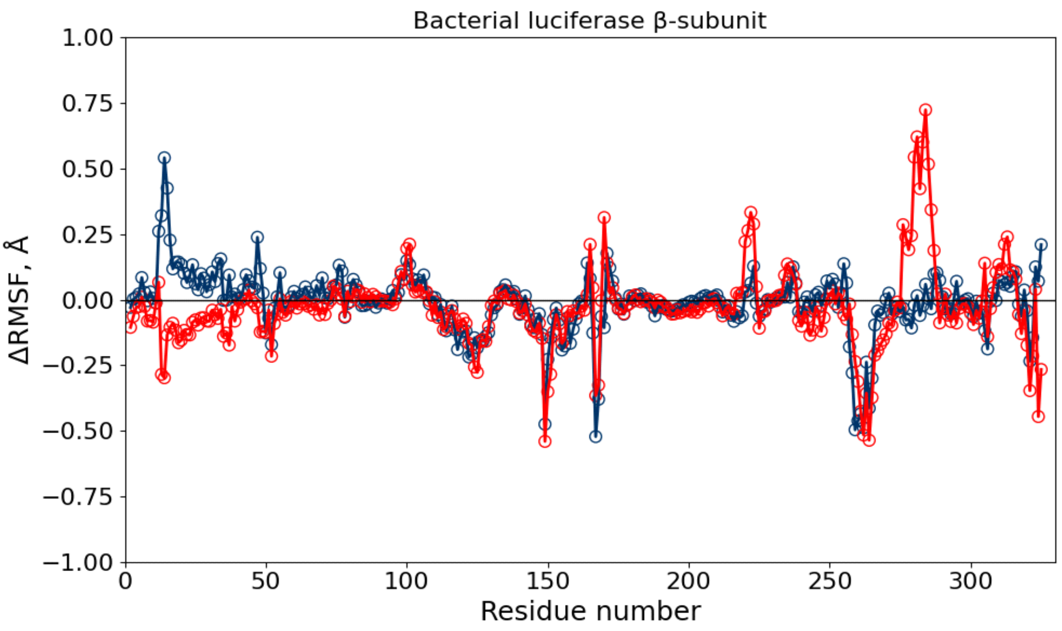

Figure S1. ΔRMSF of Cα atoms of the BLuc β-subunit in different solutions, calculated relative to the system in water. Blue empty markers show the difference between RMSF in 10% DMSO aqueous solution and water, red empty markers - between 10% DMSO aqueous solution with diesel and water. The negative ΔRMSF corresponds to a more rigid segment as compared with the structure in water.

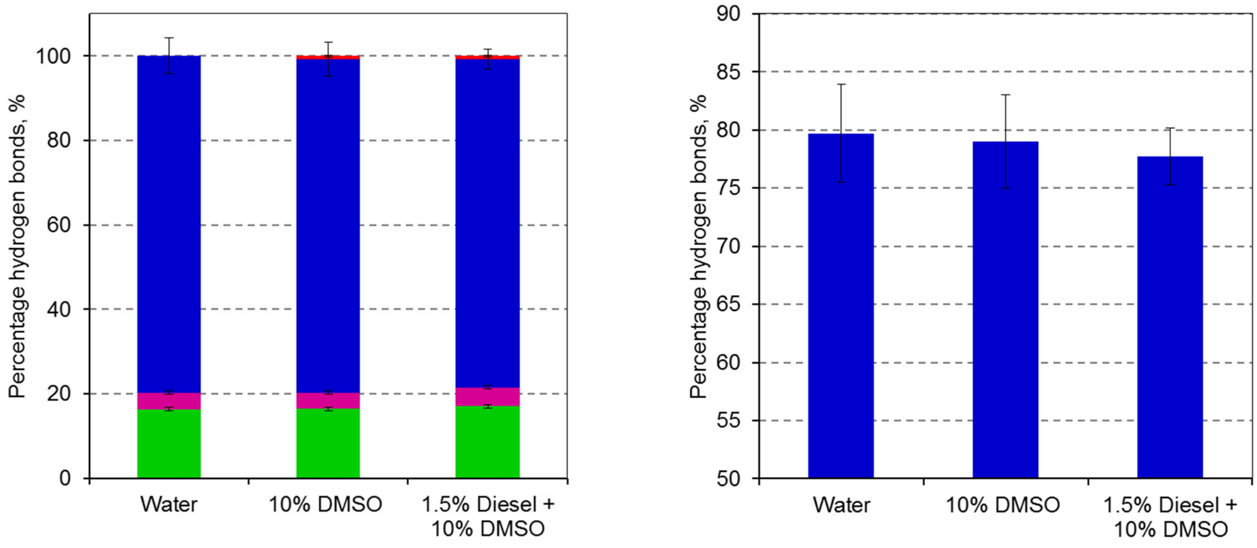

(a) (b)

Figure S2. Percentage of total hydrogen bonds for Luc (a), including the bonds between atoms of the main chain (green), sidechain (pink), protein and water (blue), protein and DMSO (red) in systems containing water, 10% DMSO, 10% DMSO and 1.5 % diesel. Panel (b) represents the percentage of hydrogen bonds between protein and water alone relative to the total number.

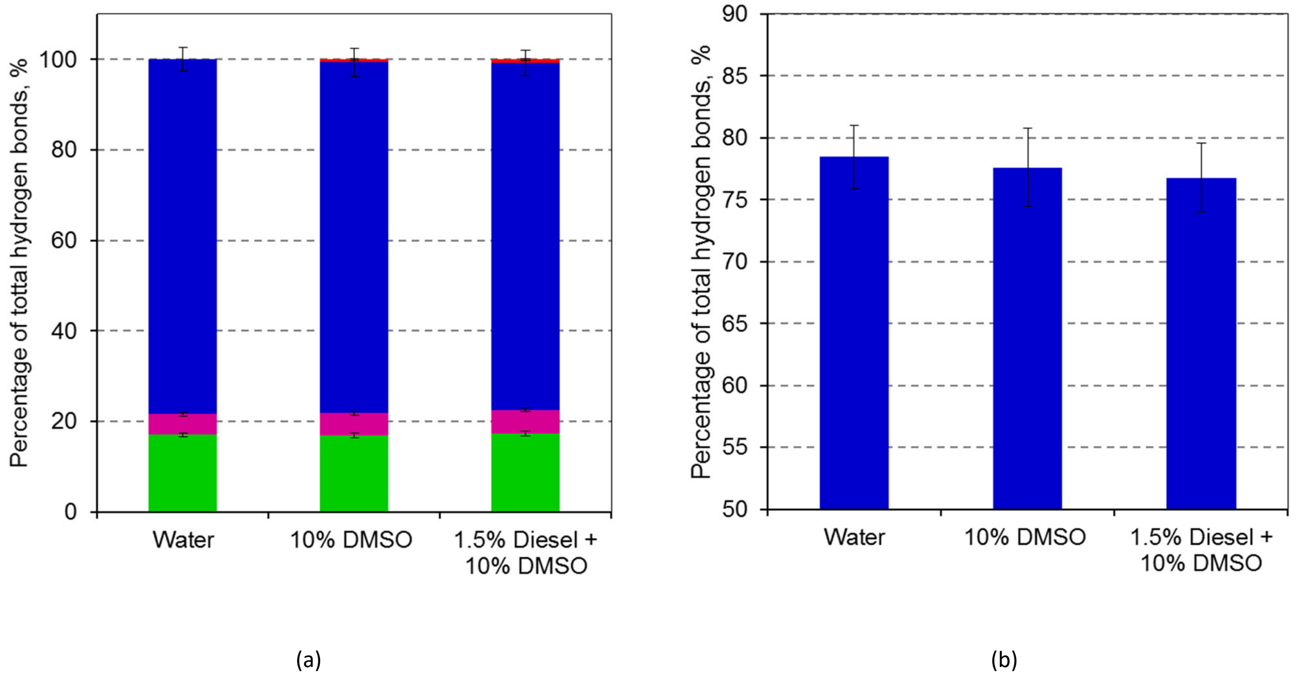

Figure S3. Percentage of total hydrogen bonds for Red (a), including the bonds between atoms of the main chain (green), sidechain (pink), protein and water (blue), protein and DMSO (red) in systems containing water, 10% DMSO, 10% DMSO and 1.5 % diesel. Panel (b) represents the percentage of hydrogen bonds between protein and water alone relative to the total number.

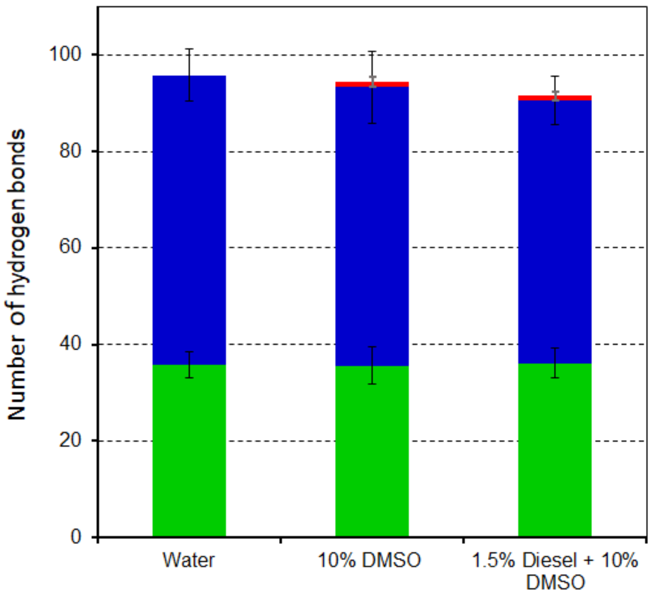

Figure S4. Number of hydrogen bonds formed by active-site residues with other protein residues (green), water (blue), and DMSO (red) in the three simulated systems for Red subunit B.
